# En face view of the transcatheter heart valve from deep right-anterior-oblique cranial position for coronary access after transcatheter aortic valve implantation: a case series

**DOI:** 10.1093/ehjcr/ytac059

**Published:** 2022-02-07

**Authors:** Suguru Hirose, Yusuke Enta, Kazunori Ishii, Arata Inoue, Masaki Nakashima, Takehiro Nomura, Makoto Saigan, Norio Tada

**Affiliations:** 1 Department of Cardiology, Sendai Kousei Hospital, 4-15 Hirosemachi Aoba Sendai, Miyagi 980-0873, Japan; 2 Department of Cardiovascular Medicine, School of Medicine, Dokkyo Medical University, Tochigi, Japan

**Keywords:** Transcatheter aortic valve replacement, En face view, Short-axis view, Fluoroscopic anatomy, Percutaneous coronary intervention, Coronary ostia access, Case series, Case report

## Abstract

**Background:**

Coronary access after transcatheter aortic valve implantation (TAVI) is challenging due to the changes in aortic geometry. The perpendicular (long-axis) view of the transcatheter heart valve (THV) is usually used as the primary fluoroscopic angle. However, it does not always provide sufficient information on the rotational axis needed for selective coronary ostia engagement. The en face (short-axis) view from the deep right-anterior-oblique cranial position gives us additional information about three-dimensional spatial relationship of the THV and coronary ostia.

**Case summary:**

We present three cases of coronary access after TAVI. We were successful in the use of the ‘en face’ view along with the perpendicular view in these cases.

**Discussion:**

The use of the en face view complements that of the perpendicular long-axis view since it allows the understanding of the three-dimensional spatial relationship of the THV and the coronary ostia during fluoroscopy and control of catheter manipulation in two directions (up/down for perpendicular and clockwise/counterclockwise for en face view). We believe that the en face view helps improve the technical success of coronary access after TAVI.

Learning points

- In coronary access after transcatheter aortic valve implantation (TAVI), the perpendicular view of the long axis is used most often as the primary fluoroscopic angle.
- We present three cases demonstrating the usefulness of the en face (short-axis) view to aid coronary access after TAVI.
- The deep right-anterior-oblique cranial position allows visualization of the en face view, which enhances understanding of the three-dimensional anatomical orientation.

## Introduction

Coronary access after transcatheter aortic valve implantation (TAVI) might have some technical issues if the transcatheter heart valve (THV) extends above the coronary ostium.^1^^,^^2^ Fluoroscopic projection angles are used to assess the three-dimensional geometric relationship between the THV and coronary ostium after placement. The perpendicular (long-axis) view of the THV is most frequently employed (*Figure 1*)^3^ but does not always provide sufficient information on the rotational axis to engage the coronary ostia. The en face (short-axis) view from the deep right-anterior-oblique (RAO) cranial position provides additional information (*Figure 1*).

**Figure 1:**
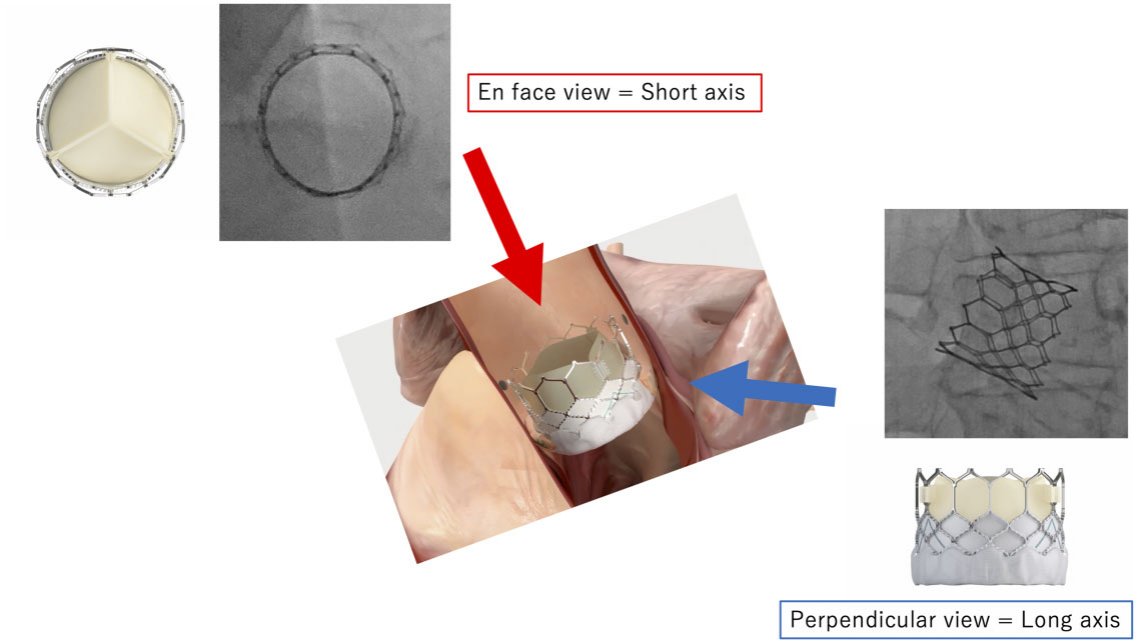
Two fluoroscopic angles for coronary access after transcatheter aortic valve implantation. The red and blue arrows indicate the angle of en face and perpendicular views, respectively.

**Figure 2:**
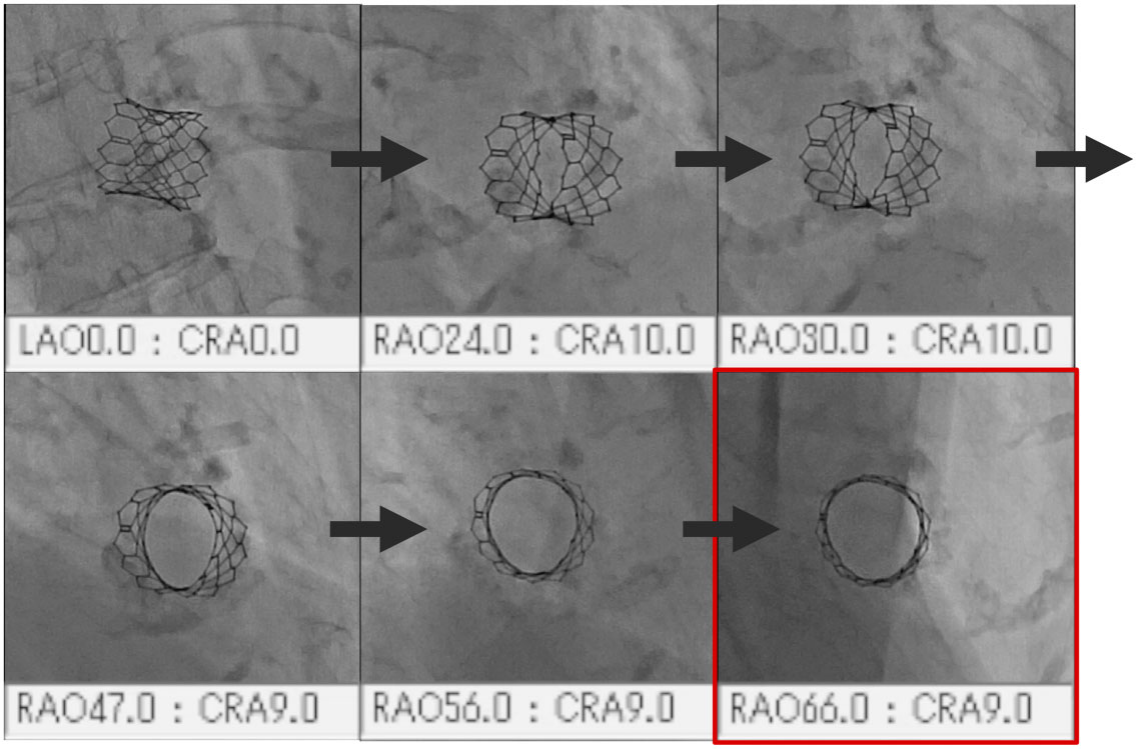
How to choose the fluoroscopic angle of the en face view. A case after SAPIEN3 20 mm implantation. The fluoroscopy views the valve frame and turns into the deep right-anterior-oblique cranial position to find a view showing the transcatheter heart valve from directly above. This case was right-anterior-oblique 66°, cranial 9° (red square). A related movie is shown in a supplemental file.

The fluoroscopic angle for the en face THV view depends on the individual patient. The optimum angle may be established by viewing the THV under fluoroscopy and turning into the deep RAO cranial position, until an optimal en face view is obtained (*Figure 2*, *Video 1*). In some cases, establishing the true en face THV view is difficult because the RAO cranial angle is too steep for the fluoroscopic machine. In these cases, an incomplete en face view still works for understanding the orientation of the short axis. Here, we present three cases for which the en face view from the deep RAO cranial angle was useful in achieving coronary access after TAVI.

## Timeline

**Table:** 

	Time	Event
Patient 1	Presentation	An 84-year-old woman with severe symptomatic aortic stenosis and a 75% stenosis of the left anterior descending artery (LAD) underwent transcatheter aortic valve implantation (TAVI) using a 23-mm SAPIEN 3 heart valve.
	Two months after TAVI	She complained of chest discomfort on effort. Exercise stress electrocardiogram showed ST depression, therefore underwent percutaneous coronary intervention (PCI) for LAD stenosis. The en face view was useful to engage the guide catheter through the cell of SAPIEN3.
	At the 6 months follow-up	She was doing well.
Patient 2	Presentation	An 89-year-old woman with severe symptomatic aortic stenosis underwent TAVI using a 20-mm SAPIEN 3 heart valve.
	One day after TAVI	The patient complained of chest pain. Electrocardiogram showed ST depression in V2–6 leads. Her creatine kinase (CK) and CK–myocardial band fraction levels were elevated. Because we had known that pre-procedural computed tomography showed that the coronary artery was normal, we suspected the coronary obstruction due to leaflet calcification. We performed emergency PCI to the left main trunk for coronary obstruction. The en face view was useful to view the proximal stent edge relative to the SAPIEN3 frame.
	5 days after PCI	The post-procedural course was uneventful and discharged.
Patient 3	Presentation	An 87-year-old man underwent TAVI using a 29-mm Evolut PRO+ heart valve.
	Two months after TAVI	He complained of shortness of breath. To assess lesion-specific ischaemia at the residual LAD stenosis, we performed coronary angiography and measured the patient’s fractional flow reserve (FFR). The en face view was useful to view a virtual triangle indicating the location of circumferential sealing skirts referring to the commissural tab. Because FFR was positive, we performed PCI.
	Three months later	He was doing well.

## Case presentation

### Patient 1

**Figure 3:**
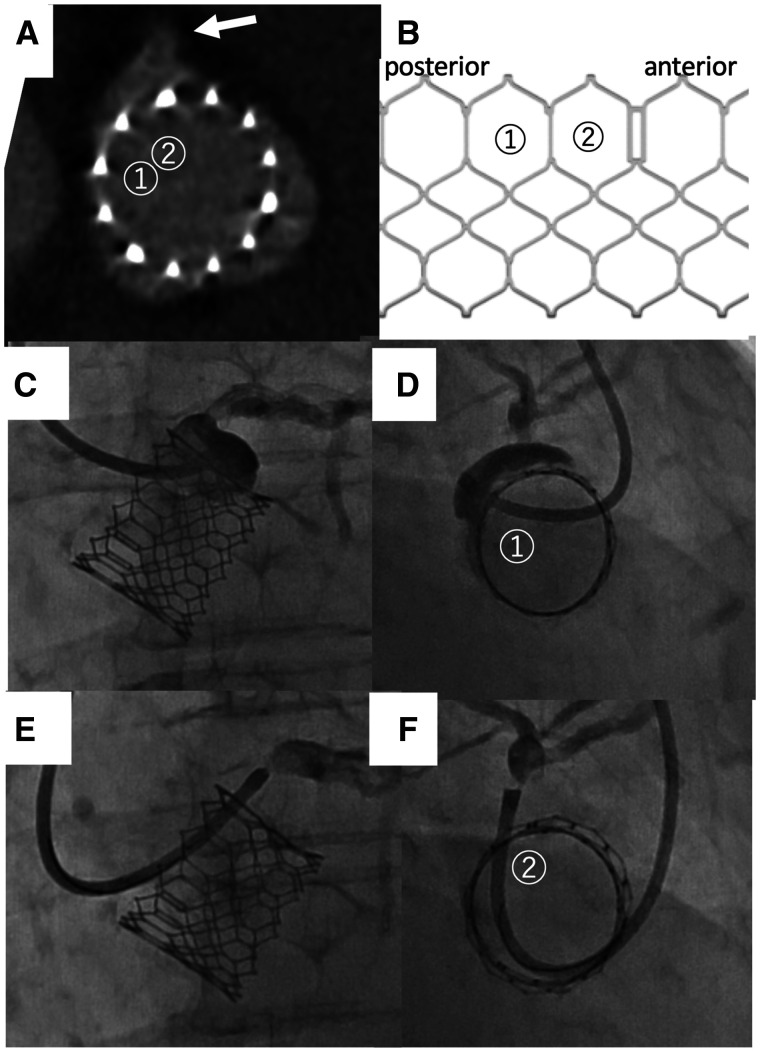
Patient 1: Appropriate selection of transcatheter heart valve cells under guidance of the en face view using the deep right-anterior-oblique cranial position (right-anterior-oblique 71°, cranial 35°) to achieve selective coronary engagement. (*A*) Short-axis computed tomography image after transcatheter aortic valve implantation. The arrow indicates the left main coronary artery. (*B*) The SAPIEN 3 frame. A 6-Fr ASAHI Hyperion Super Power Backup 3.0 catheter was selected as the guide catheter from the left radial artery. ① indicates the cell crossed in (*C* and *D*). ② indicates the cell crossed in (*E* and *F*). On the first attempt, the guide catheter was crossed through cell ①. Angiograms taken from the perpendicular (*C*) and the en face views (*D*) simultaneously. The en face view (*D*) shows the guide catheter passing through the cell posterior to the left main coronary artery. On the second attempt, the catheter was passed through cell ② in front of the LMCA ostia (*E* and *F*).

An 84-year-old Asian woman was referred to our hospital for treatment of severe symptomatic aortic stenosis and a 75% stenosis of the left anterior descending artery (LAD). Systolic murmur was audible. Our heart team decided to perform TAVI using a 23 mm SAPIEN 3 heart valve and (Edwards Lifesciences, Irvine, CA, USA) (*Figure 3B*) then observe the disease course before deciding whether to perform further intervention.

Two months after TAVI, the patient began to experience chest discomfort on effort. Exercise stress electrocardiogram showed ST depression. Echocardiography showed no abnormal THV function. Therefore, we performed a percutaneous coronary intervention (PCI) to treat the LAD stenosis from her left radial artery. Computed tomography performed after TAVI is shown in *Figure 3A*. A 6 Fr ASAHI Hyperion Super Power Backup 3.0 catheter (ASAHI INTECC CO LTD, Aichi, Japan) was chosen as the guide catheter. We cannulated the left main coronary artery (LMCA) through the upper row of cells in the THV frame guided by the perpendicular fluoroscopic view (*Figure 3C*). The catheter crossed into the left sinus but the selective engagement was not achieved.^2^ Therefore, we consulted the deep RAO cranial en face view (RAO 71°, cranial 35°) (*Figure 3D*). This orientation revealed that the catheter had passed through the cell posterior to the LMCA (Figure *3**A* and *B*). We used a clockwise rotation to cross the anterior cell, which allowed selective coronary engagement and completion of the PCI (*Figure 3E* and *F*). At the 6 months of follow-up, she was doing well.

### Patient 2

**Figure 4:**
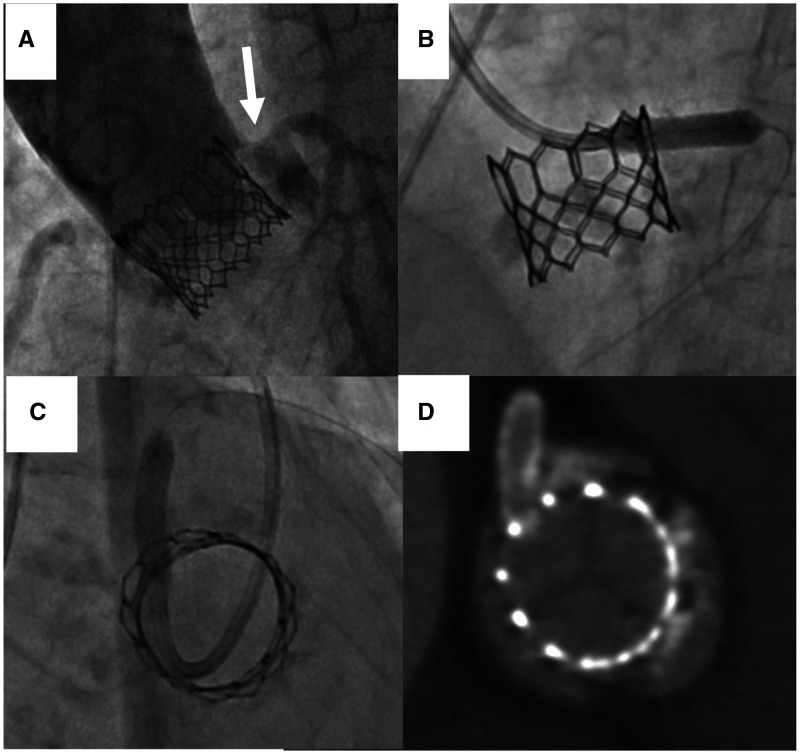
Patient 2: The usefulness of the en face short-axis view for visualizing the positional relationship between the coronary stent and the SAPIEN 3 frame. A left coronary occlusion occurred after SAPIEN 3 implantation. We planned to implant the proximal edge of the stent directly against the SAPIEN 3 frame using a 6 Fr ASAHI Hyperion Judkins Left 3.5 catheter from the left radial artery. The aortogram (*A*) shows calcified native valve tissues occupying the left sinus (arrow). (*B* and *C*) Fluorographic images taken during coronary stent implantation in the perpendicular (*B*) and the en face views (*C*) simultaneously. In contrast to the perpendicular view (*B*), the en face view (*C*) from the deep right-anterior-oblique cranial position (right-anterior-oblique 60°, cranial 42°) shows the positional relationship between the coronary stent and SAPIEN 3 frame. (*D*) Computed tomography imaging after percutaneous coronary intervention shows that the stent was implanted as planned.

An 89-year-old Asian woman with symptomatic severe aortic stenosis underwent TAVI using a 20 mm SAPIEN 3 heart valve. Systolic murmur was audible before TAVI. Even though a coronary height cut-off <10 mm increases the risk of coronary obstruction during TAVI,^4^ her left coronary height was low as at 9.8 mm. Aortography after THV implantation showed the excessive leaflet calcification occupied the left sinus. The coronary flow was not delayed, therefore we assessed that coronary obstruction had not occurred (*Figure 4A*). However, 1 day after the procedure, the patient complained of chest pain. Electrocardiogram showed ST depression in V2–6 leads. Her creatine kinase (CK) (the upper limit of normal was 199 U/L in females) and CK–myocardial band fraction levels were elevated to 355 and 54 U/L, respectively. Because we had known that pre-procedural computed tomography showed that the coronary artery was normal, we suspected the coronary obstruction due to leaflet calcification (*Figure 4A*). Emergency PCI to the LMCA was performed from her left radial artery. After achieving coaxial cannulation into the left coronary artery using a 6 Fr ASAHI Hyperion Judkins Left 3.5 catheter (ASAHI INTECC CO LTD, Aichi, Japan), we performed the intravascular ultrasound showing that the calcified aortic valve leaflet occupied the left coronary ostium. Thus, we diagnosed coronary obstruction after SAPIEN3 implantation. We planned to implant the proximal edge of the stent directly against the SAPIEN 3 frame. From the perpendicular view, we were unable to assess the correct the stent position (*Figure 4B*). Then, we used the en face view in the deep RAO cranial position (RAO 60°, cranial 42°) to view the proximal stent edge relative to the THV frame (*Figure 4C*). Computed tomography after PCI revealed that the stent was implanted as planned (*Figure 4D*). The post-procedural course was uneventful and discharged 5 days later.

### Patient 3

**Figure 5:**
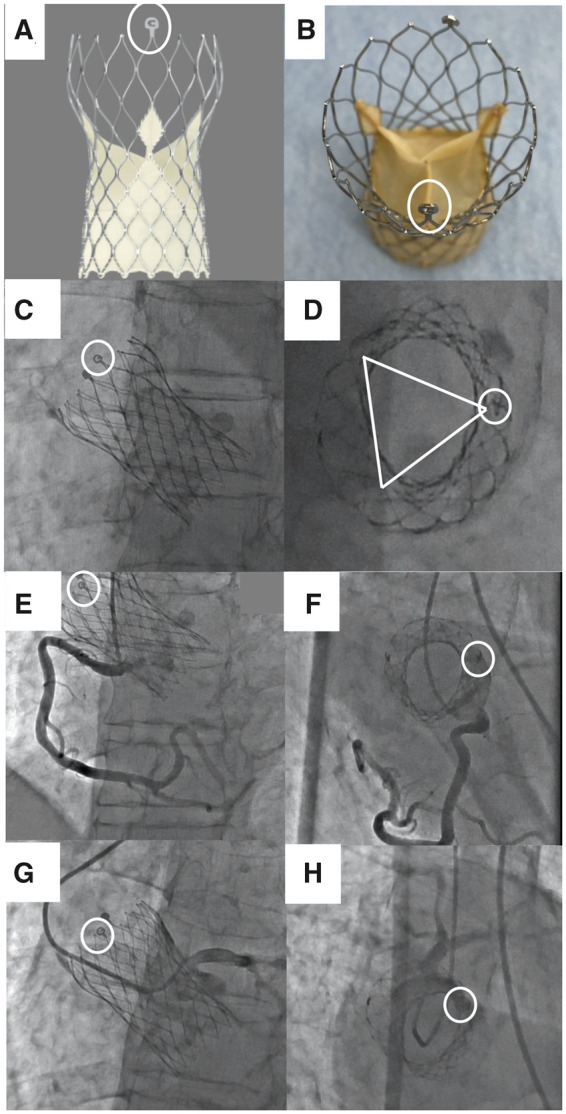
Patient 3: Use of the commissure tab on the Evolut PRO+ transcatheter heart valve to visualize the appropriate location via which to engage the coronary ostia. White circles indicate the commissural tab (C-tab) (*A–H*). (*C, E,* and *G*) Perpendicular images. (*D, F,* and *H*) Right-anterior-oblique cranial en face short-axis images (right-anterior-oblique 74°, cranial 48°). From the commissure tab position, a virtual triangle was constructed to indicate the location of three commissure positions on the short-axis image (*D*). By avoiding the apex of the virtual triangle, engaging the coronary ostia became much easier and enabled right (*E* and *F*) and left (*G* and *H*) coronary angiography using 5Fr JR4 and JL4 diagnostic catheters from the left radial artery.

An 87-year-old Asian man with symptomatic severe aortic stenosis underwent TAVI with a 29 mm Evolut PRO + heart valve (Medtronic, Minneapolis, MN, USA). Systolic murmur was heard before TAVI. We were aware of a 75% stenosis of the LAD; however, we managed this conservatively with a view to monitoring him clinically after TAVI for evidence of ischaemia. He complained of shortness of breath 2 months later. His chest X-ray did not show any abnormal change and his echocardiography showed normal left ventricle and THV function. We performed coronary angiography and fractional flow reserve (FFR) to assess lesion-specific ischaemia. 5 Fr JR4 and JL4 diagnostic catheters were used from his left radial artery. The Evolut series THVs have circumferential sealing skirts that extend upward toward the commissural insertion point (Figure *5**A* and *B*) through which the catheter cannot easily cross.^1^ The commissural tab (C-tab) on the THV frame provides a marker for the location of one of the commissures (*Figure 5A‒C*). When referring to this C-tab from the deep RAO cranial position (RAO 74°, cranial 48°), a virtual triangle indicating the location of the other THV commissures on fluoroscopy can be drawn (*Figure 5D*). By avoiding the triangle apex, engaging the ostia became much easier (*Figure 5F,H*). As a result, we successfully performed right and left coronary angiography and FFR in this patient (*Figure 5E–H*). Fractional flow reserve with intracoronary nicorandil injection was 0.79 at the 75% stenosis of the LAD, therefore we performed PCI. At the 3 months follow-up after PCI, he was doing well.

## Discussion

In this case series, we present three cases that demonstrate the usefulness of the en face view of THVs when used in combination with the perpendicular view. The latter is used most often as the primary fluoroscopic angle to guide coronary ostia access after TAVI.^3^ The en face view from the deep RAO cranial position provides additional information about the three-dimensional anatomical orientation of the THV and coronary ostia and helps to clarify the appropriate rotational axis needed to control the catheter in multiple directions. This was helpful in Patients 1 and 3. Particularly, we should torque the catheter clockwise to engage the right coronary artery (RCA) as shown in Patient 3. The RE-ACCESS study^2^ reported that semi-selective cannulation was observed more in the RCA than in the left coronary artery. This finding might have been due to difficulty in crossing through the THV frame cells coaxial to the coronary ostia because the rotational axis was not clear. As in the cases we describe, the use of biplane fluoroscopy to visualize the left-anterior-oblique perpendicular and RAO cranial en face views simultaneously can be extremely useful in these cases. We believe that the use of the en face view would improve the rate of the selective coronary engagement after TAVI. This approach should be assessed further in larger studies.

The fluoroscopic angle showing the true en face view varies between patients. In the three cases presented, deep angles were required to obtain the optimal view. The relevant angle might also depend on the orientation of THV deployment. A further study is required to assess the number of patients in whom the optimal en face view may be achieved given the limitations of existing fluoroscopy systems.

One potential limitation of this method could be increased radiation exposure since the extreme fluoroscopic angle used requires a larger dose of radiation for the X-rays to travel the longer distance through the tissue. Whenever possible, physicians should strive to reduce radiation exposure by taking actions such as shortening the fluoroscopy time, etc.

## Conclusions

We present three cases demonstrating that the en face view of THV can improve the technical success of coronary artery access after TAVI. Use of both views informs a manipulation in two directions—up/down for perpendicular and clockwise/counterclockwise for en face view. We believe this approach has potential applications beyond those presented in this report such as standard angiography where cannulation is very difficult. 

## Lead author biography

**Figure 1:**
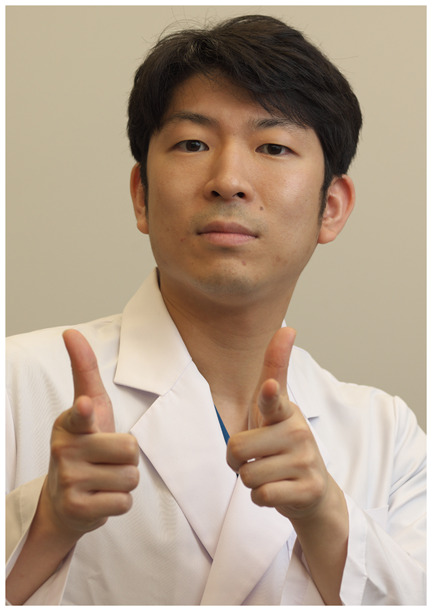


Suguru Hirose, MD, PhD, graduated from Dokkyo Medical University, Japan. He began clinical training in Cardiology at Dokkyo Medical University Hospital, Tochigi, Japan. He currently works as an interventional Cardiologist in Sendai Kousei Hospital, Japan.

## Supplementary material

Supplementary material is available at *European Heart Journal - Case Reports* online.

## Slide sets

A fully edited slide set detailing this case and suitable for local presentation is available online as Supplementary data.

## Consent

The authors confirm that written consent for submission and publication of this case series including images and associated texts has been obtained from all patients in line with COPE guidance.

## Funding

None declared.

**Conflict of interest**: N.T. is a clinical proctor for Edwards Lifesciences and Medtronic. All the other authors have no conflict of interest to disclose.

## Supplementary Material

ytac059_Supplementary_Data
