# Supplementary material for: En face view of the transcatheter heart valve from deep right-anterior-oblique cranial position for coronary access after transcatheter aortic valve implantation: a case series
Source: Eur Heart J Case Rep. 2022 Feb 7;6(2):ytac059. doi: 10.1093/ehjcr/ytac059 (PMC8874809; doi:10.1093/ehjcr/ytac059)
Supplement: ytac059_Supplementary_Data [file ytac059_Supplementary_Data.pptx]

## Slide 1
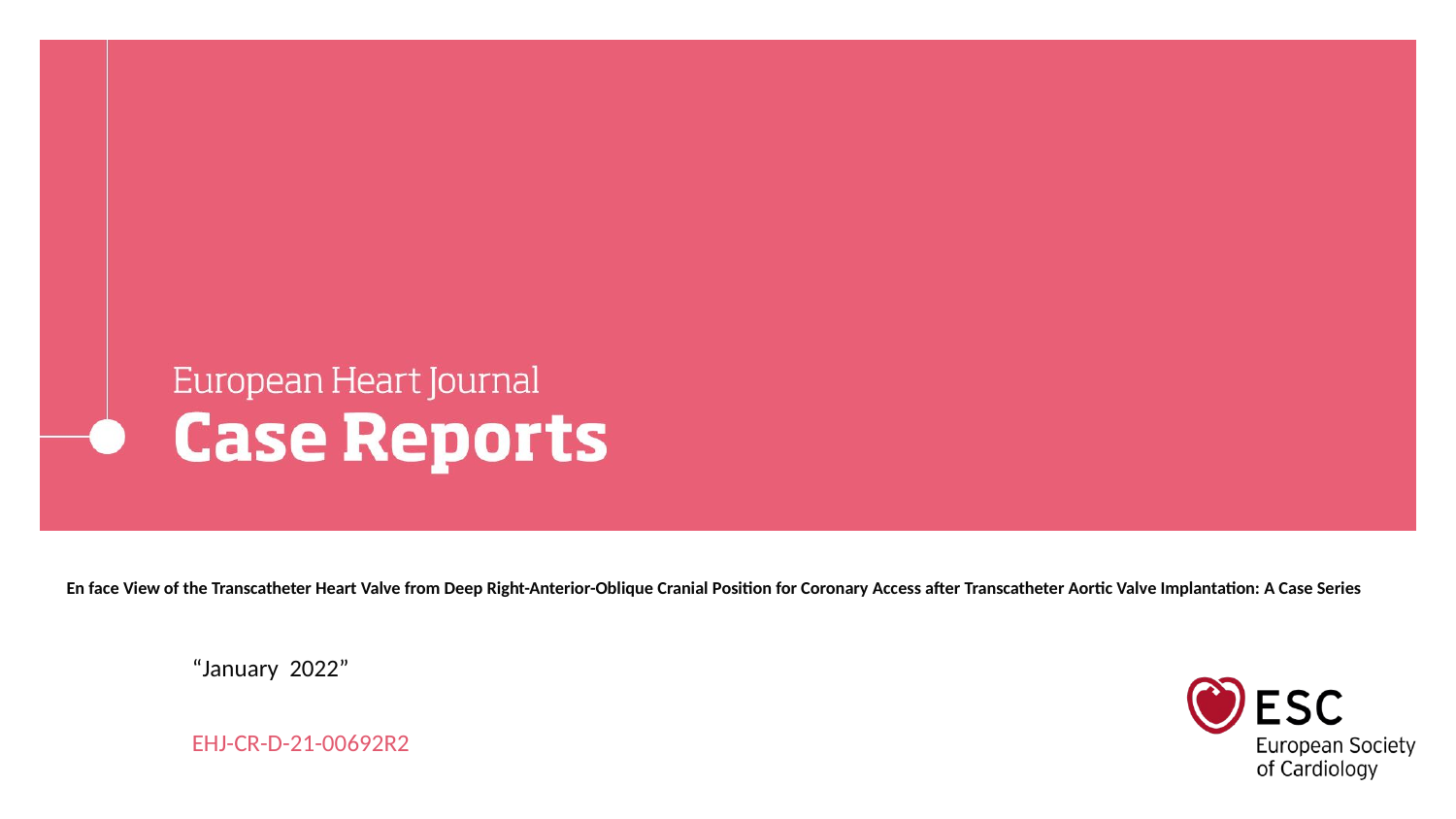

# En face View of the Transcatheter Heart Valve from Deep Right-Anterior-Oblique Cranial Position for Coronary Access after Transcatheter Aortic Valve Implantation: A Case Series
“January 2022”
EHJ-CR-D-21-00692R2

## Slide 2
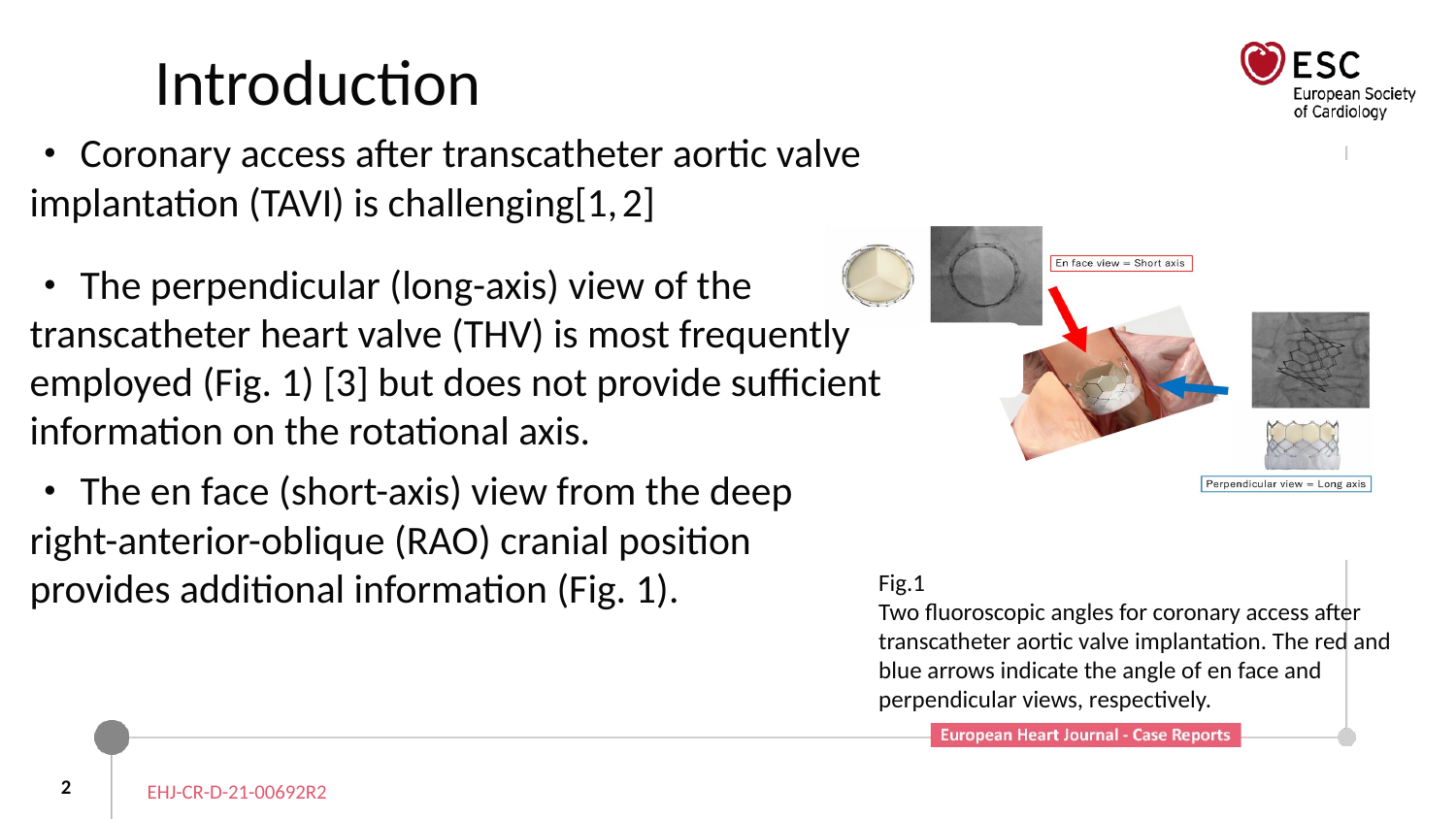

# Introduction
・Coronary access after transcatheter aortic valve implantation (TAVI) is challenging[1, 2]
・The perpendicular (long-axis) view of the transcatheter heart valve (THV) is most frequently employed (Fig. 1) [3] but does not provide sufficient information on the rotational axis.
・The en face (short-axis) view from the deep right-anterior-oblique (RAO) cranial position provides additional information (Fig. 1).
Fig.1
Two fluoroscopic angles for coronary access after transcatheter aortic valve implantation. The red and blue arrows indicate the angle of en face and perpendicular views, respectively.
EHJ-CR-D-21-00692R2
2

## Slide 3
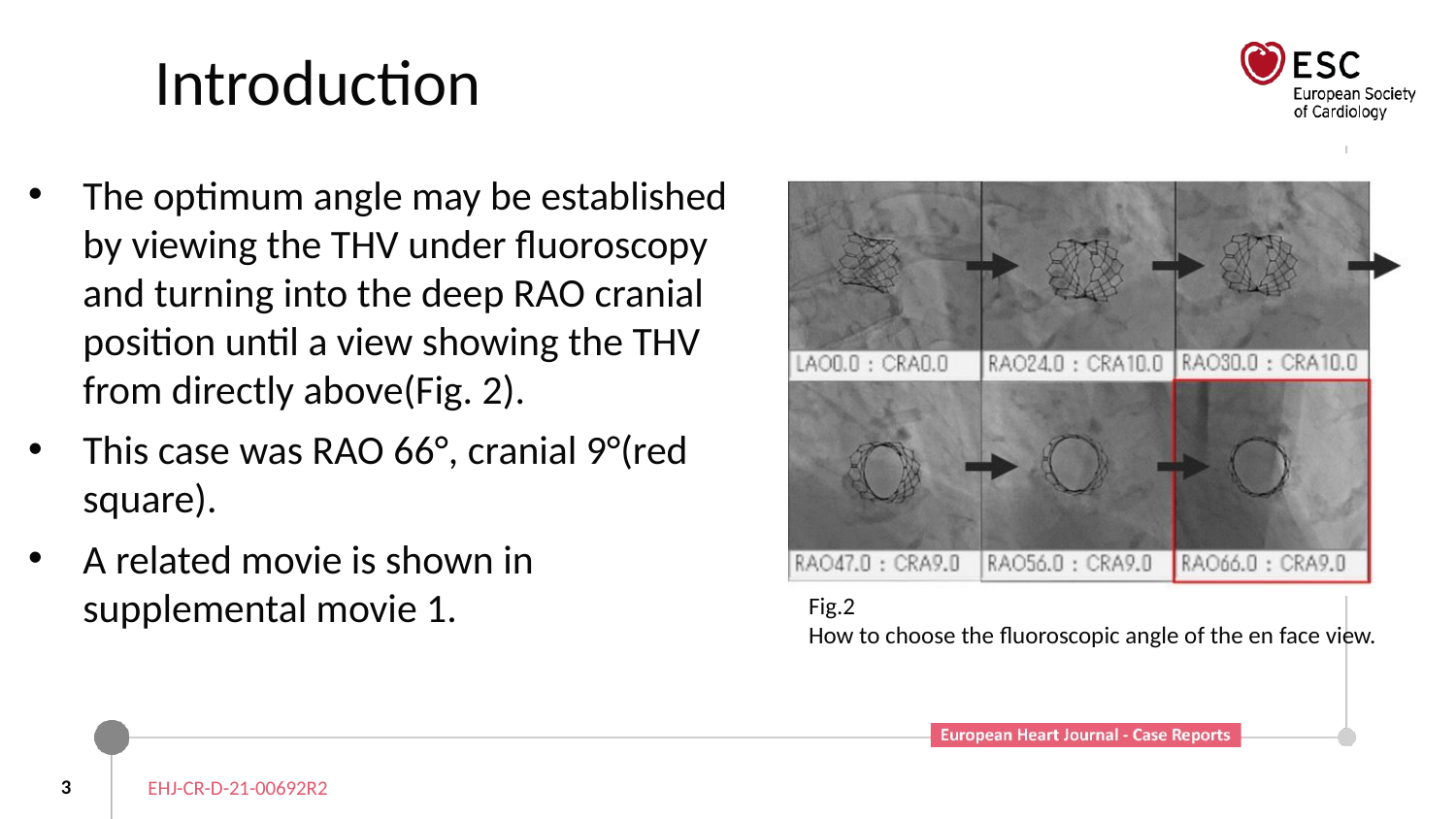

# Introduction
The optimum angle may be established by viewing the THV under fluoroscopy and turning into the deep RAO cranial position until a view showing the THV from directly above(Fig. 2).
This case was RAO 66°, cranial 9°(red square).
A related movie is shown in supplemental movie 1.
Fig.2
How to choose the fluoroscopic angle of the en face view.
3
EHJ-CR-D-21-00692R2

## Slide 4
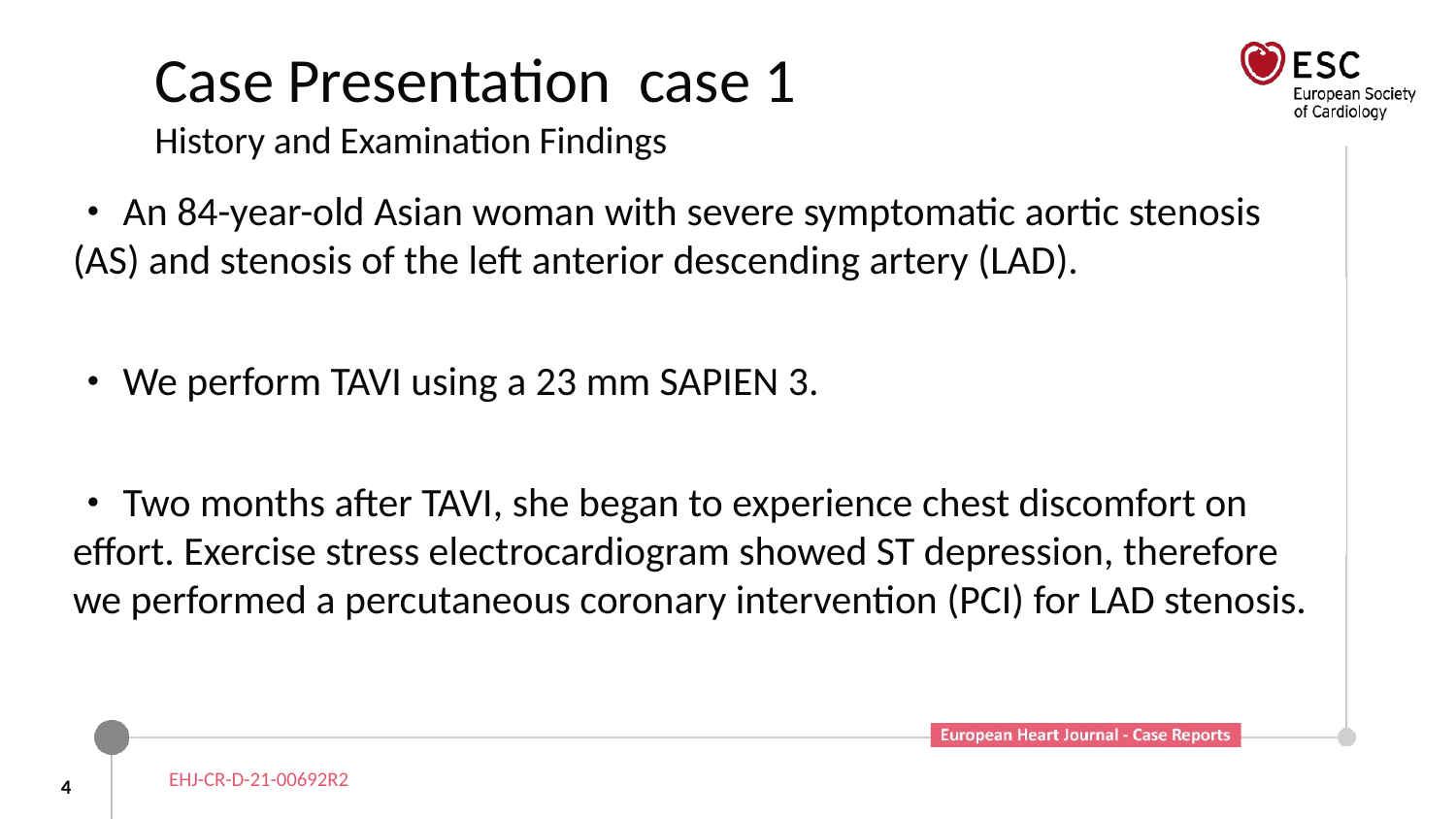

# Case Presentation case 1 History and Examination Findings
・An 84-year-old Asian woman with severe symptomatic aortic stenosis (AS) and stenosis of the left anterior descending artery (LAD).
・We perform TAVI using a 23 mm SAPIEN 3.
・Two months after TAVI, she began to experience chest discomfort on effort. Exercise stress electrocardiogram showed ST depression, therefore we performed a percutaneous coronary intervention (PCI) for LAD stenosis.
EHJ-CR-D-21-00692R2
4

## Slide 5
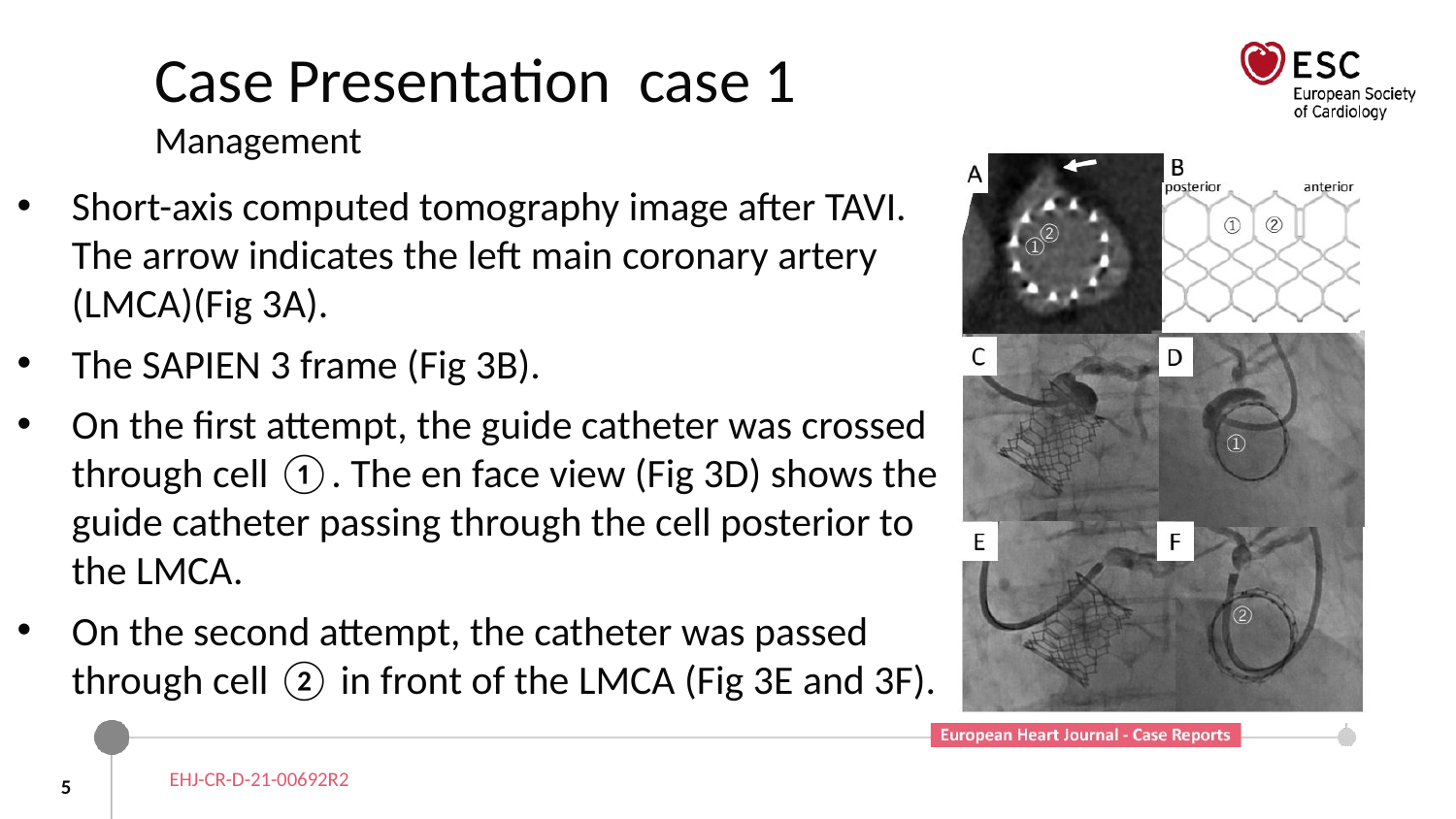

# Case Presentation case 1 Management
Short-axis computed tomography image after TAVI. The arrow indicates the left main coronary artery (LMCA)(Fig 3A).
The SAPIEN 3 frame (Fig 3B).
On the first attempt, the guide catheter was crossed through cell ①. The en face view (Fig 3D) shows the guide catheter passing through the cell posterior to the LMCA.
On the second attempt, the catheter was passed through cell ② in front of the LMCA (Fig 3E and 3F).
EHJ-CR-D-21-00692R2
5

## Slide 6
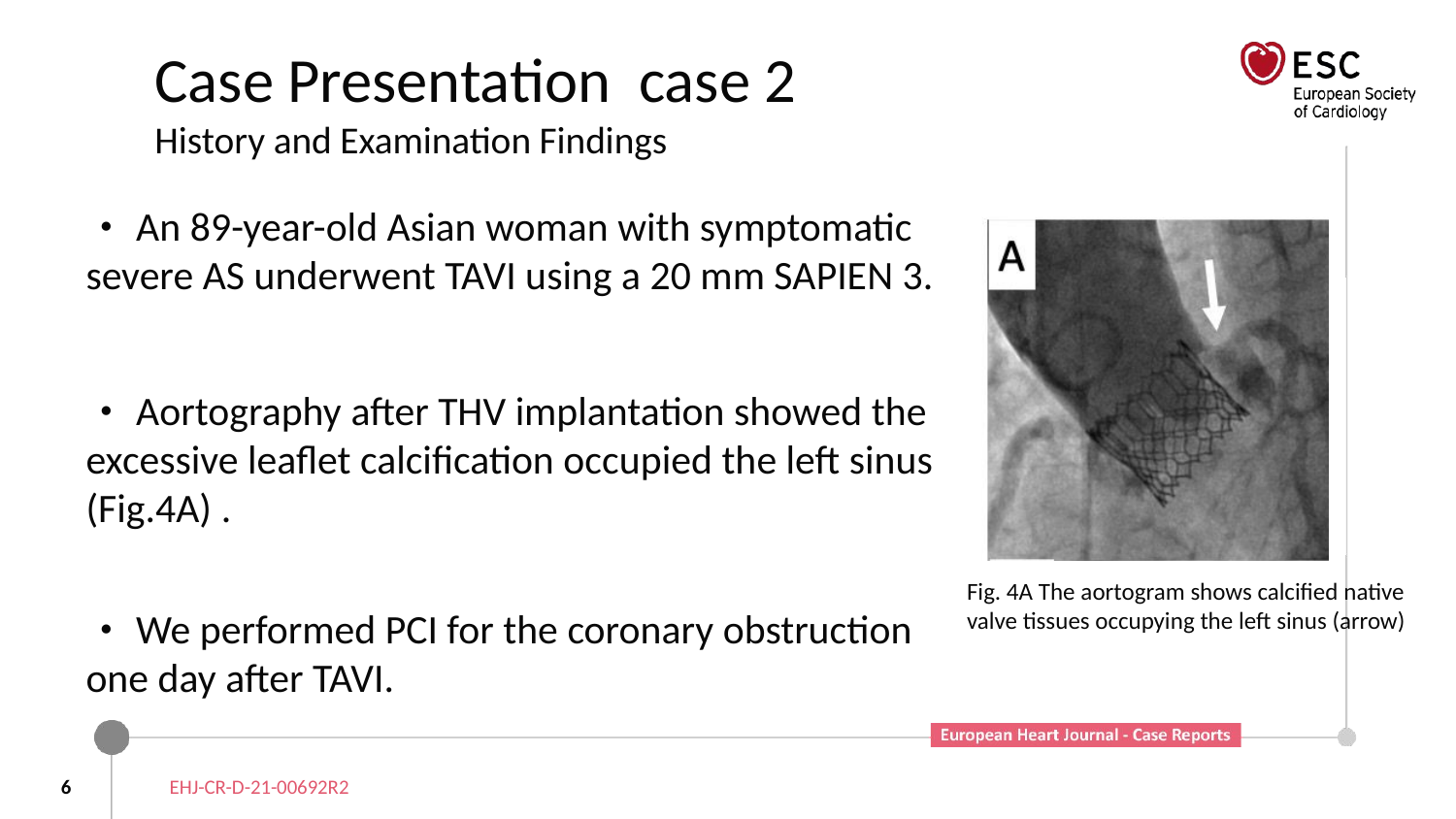

# Case Presentation case 2 History and Examination Findings
・An 89-year-old Asian woman with symptomatic severe AS underwent TAVI using a 20 mm SAPIEN 3.
・Aortography after THV implantation showed the excessive leaflet calcification occupied the left sinus (Fig.4A) .
・We performed PCI for the coronary obstruction one day after TAVI.
Fig. 4A The aortogram shows calcified native valve tissues occupying the left sinus (arrow)
6
EHJ-CR-D-21-00692R2

## Slide 7
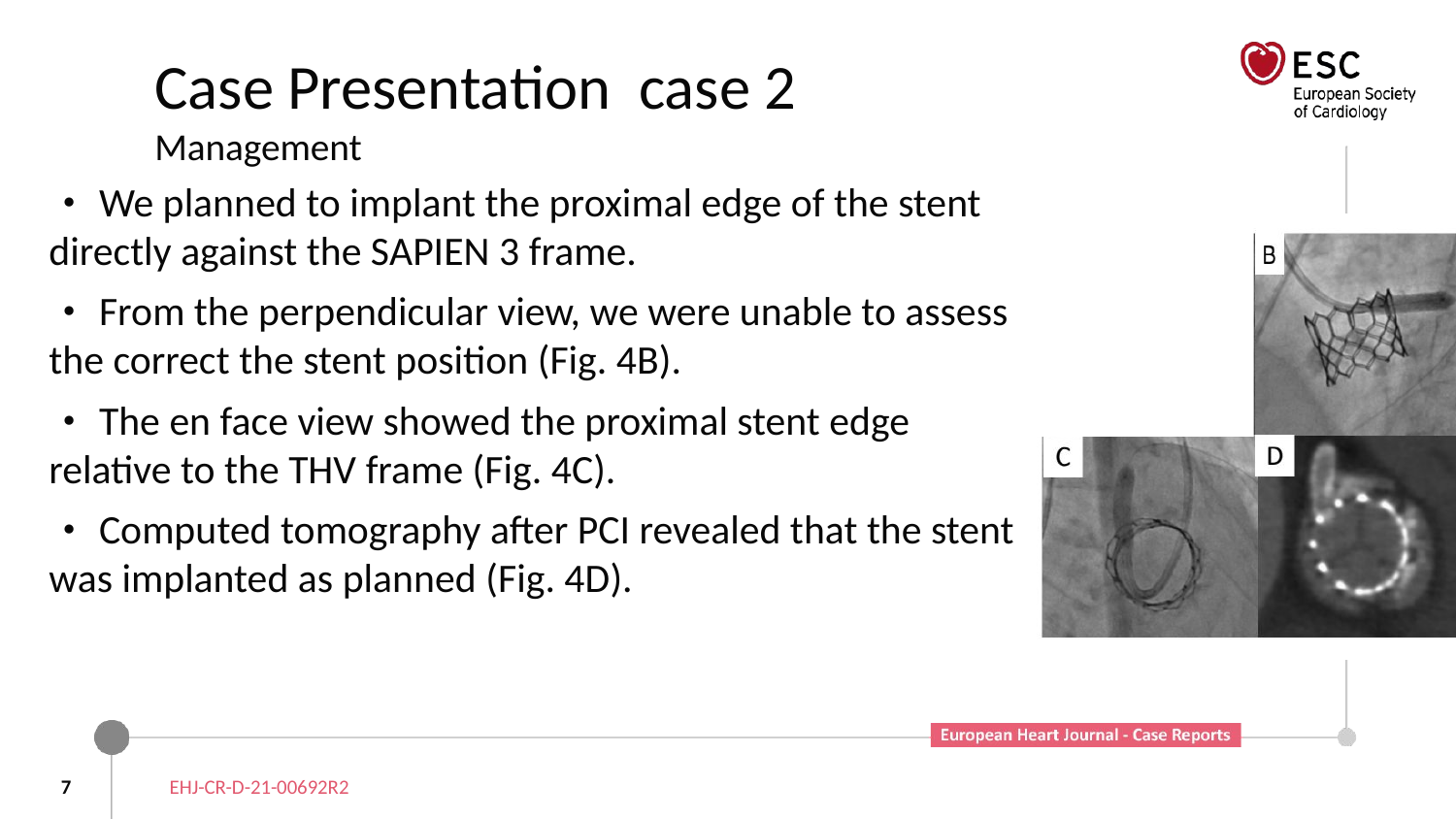

# Case Presentation case 2 Management
・We planned to implant the proximal edge of the stent directly against the SAPIEN 3 frame.
・From the perpendicular view, we were unable to assess the correct the stent position (Fig. 4B).
・The en face view showed the proximal stent edge relative to the THV frame (Fig. 4C).
・Computed tomography after PCI revealed that the stent was implanted as planned (Fig. 4D).
7
EHJ-CR-D-21-00692R2

## Slide 8
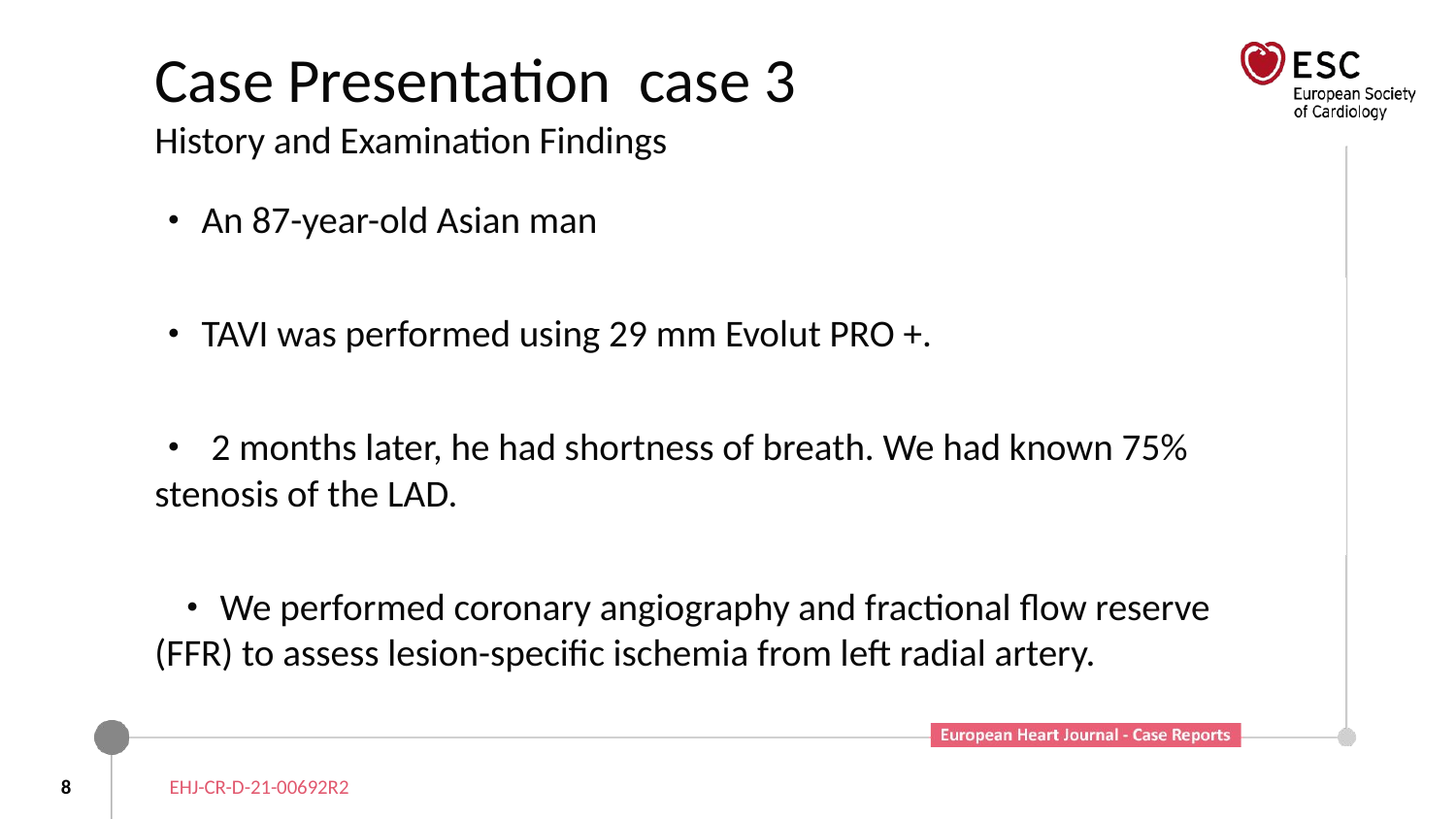

# Case Presentation case 3 History and Examination Findings
・An 87-year-old Asian man
・TAVI was performed using 29 mm Evolut PRO +.
・ 2 months later, he had shortness of breath. We had known 75% stenosis of the LAD.
 ・We performed coronary angiography and fractional flow reserve (FFR) to assess lesion-specific ischemia from left radial artery.
8
EHJ-CR-D-21-00692R2

## Slide 9
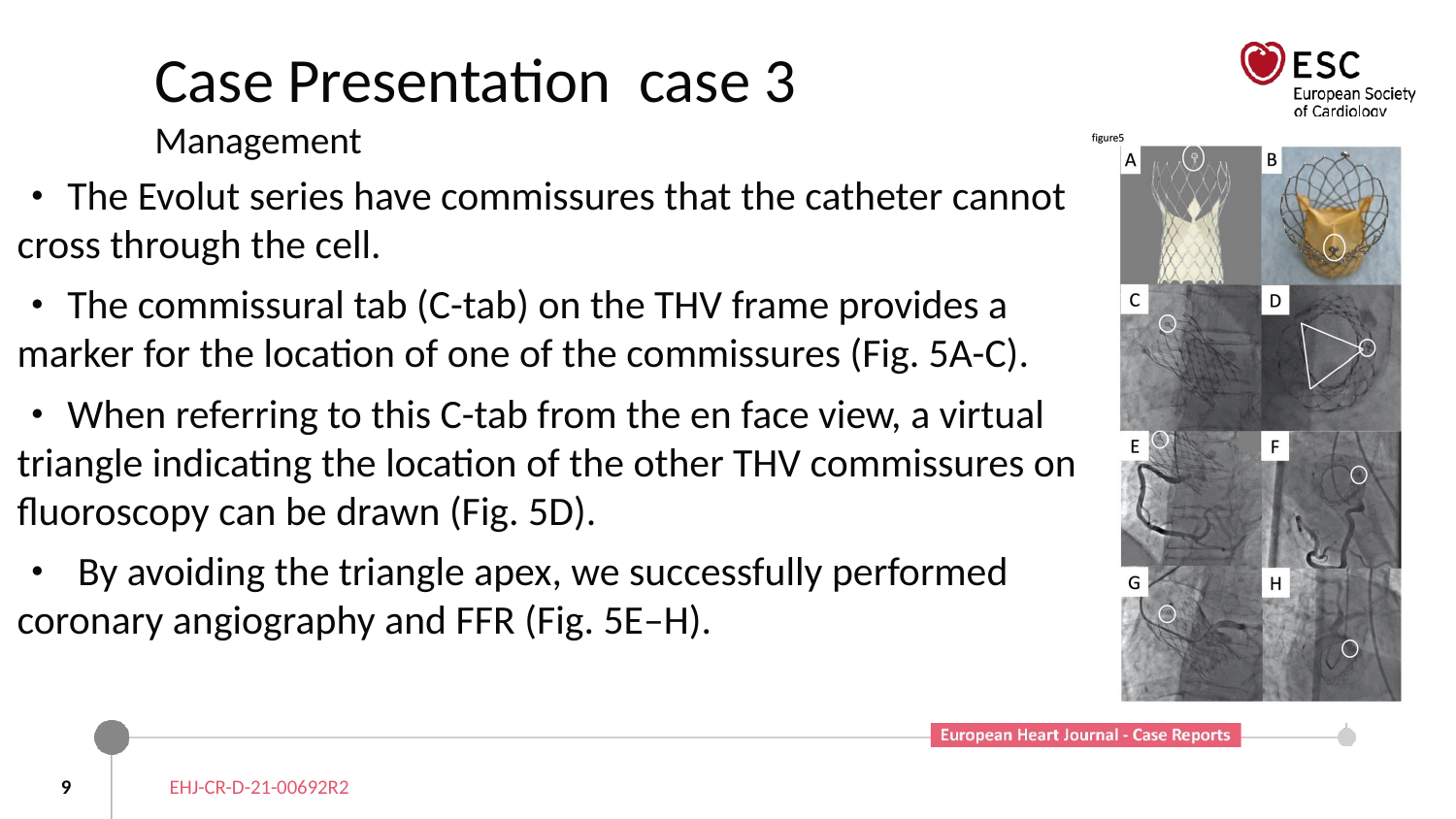

# Case Presentation case 3 Management
・The Evolut series have commissures that the catheter cannot cross through the cell.
・The commissural tab (C-tab) on the THV frame provides a marker for the location of one of the commissures (Fig. 5A-C).
・When referring to this C-tab from the en face view, a virtual triangle indicating the location of the other THV commissures on fluoroscopy can be drawn (Fig. 5D).
・ By avoiding the triangle apex, we successfully performed coronary angiography and FFR (Fig. 5E–H).
9
EHJ-CR-D-21-00692R2

## Slide 10
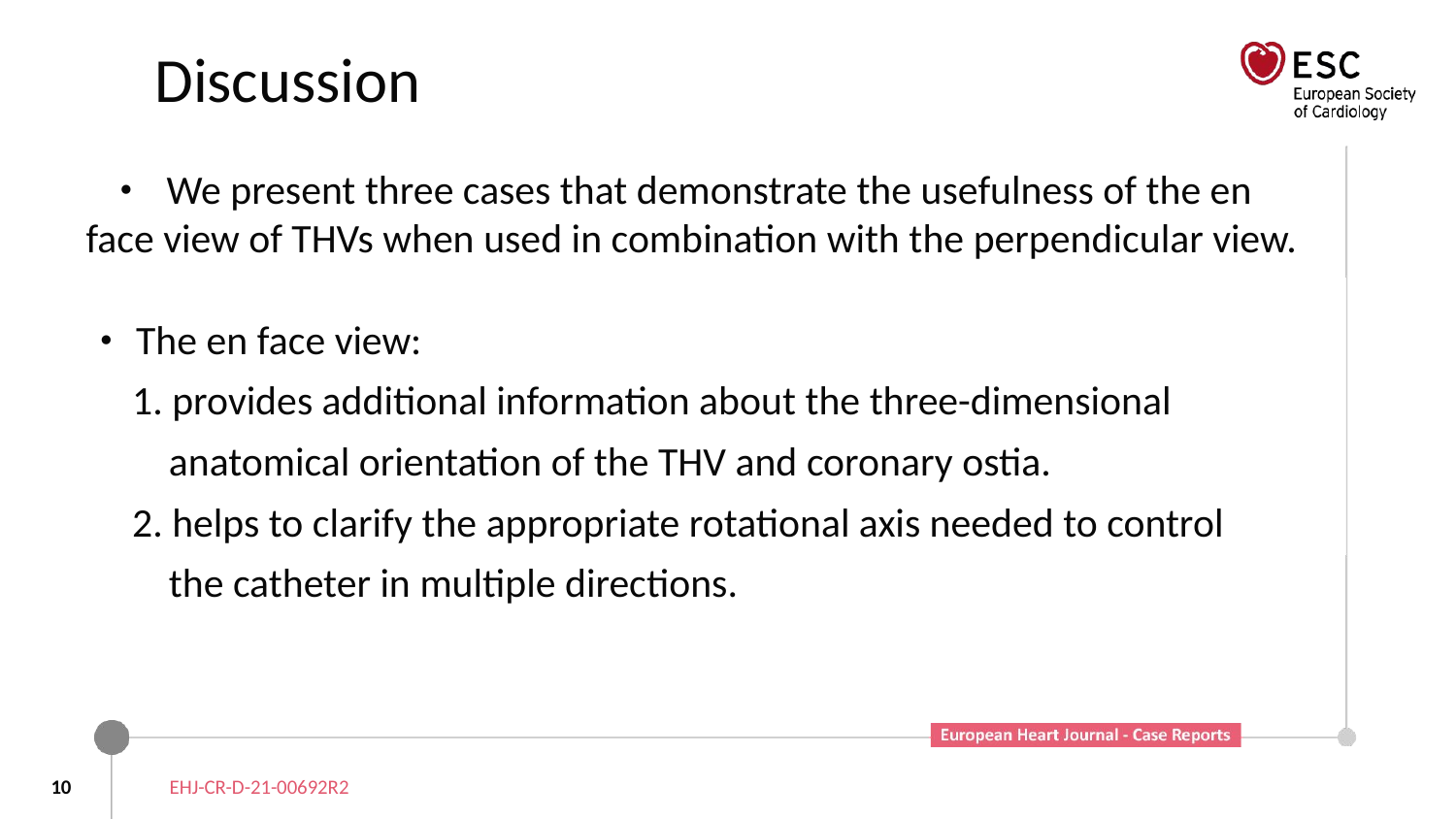

# Discussion
 ・ We present three cases that demonstrate the usefulness of the en face view of THVs when used in combination with the perpendicular view.
・The en face view:
 1. provides additional information about the three-dimensional
 anatomical orientation of the THV and coronary ostia.
 2. helps to clarify the appropriate rotational axis needed to control
 the catheter in multiple directions.
10
EHJ-CR-D-21-00692R2

## Slide 11
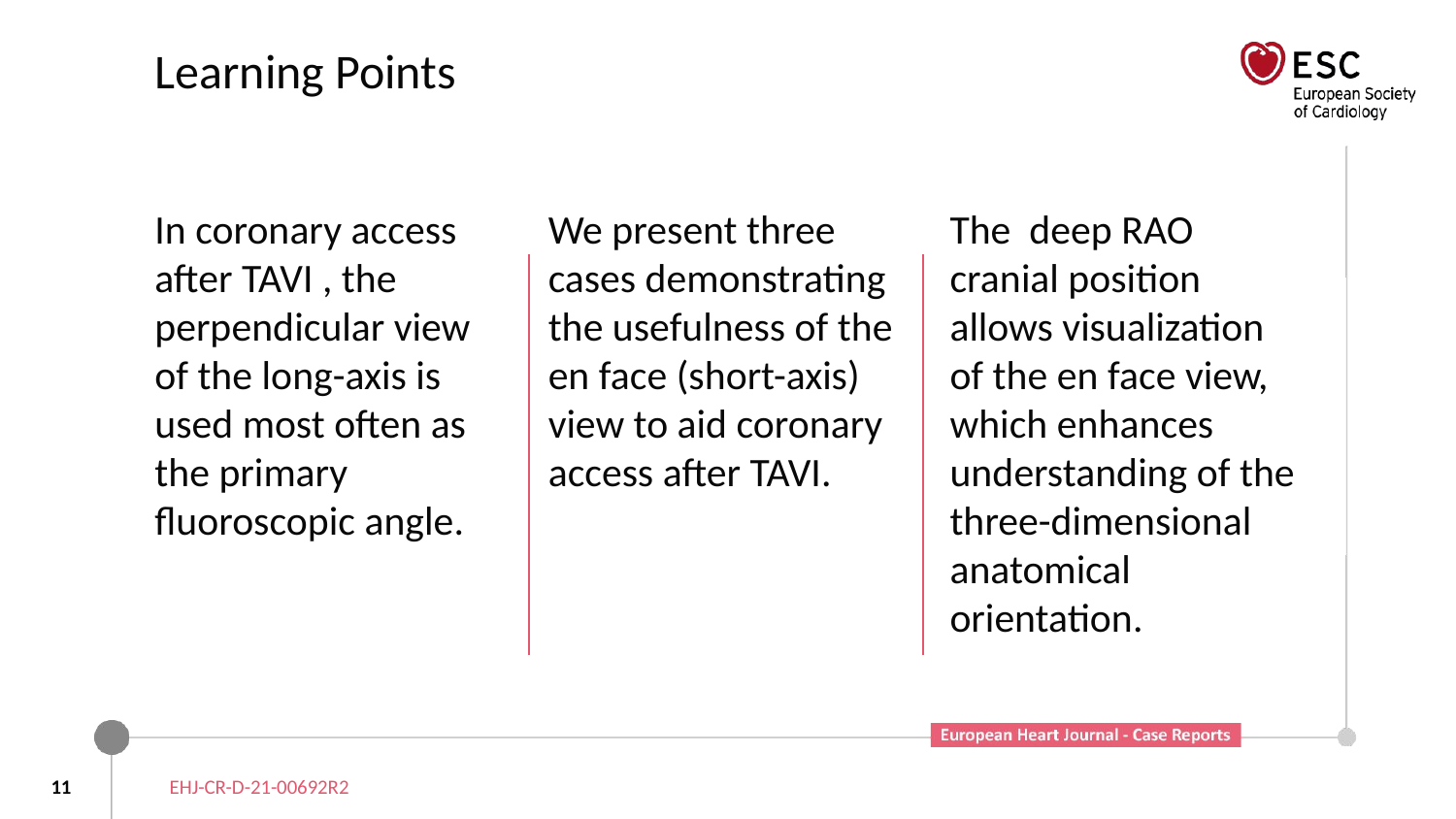

# Learning Points
In coronary access after TAVI , the perpendicular view of the long-axis is used most often as the primary fluoroscopic angle.
We present three cases demonstrating the usefulness of the en face (short-axis) view to aid coronary access after TAVI.
The  deep RAO cranial position allows visualization of the en face view, which enhances understanding of the three-dimensional anatomical orientation.
11
EHJ-CR-D-21-00692R2

## Slide 12
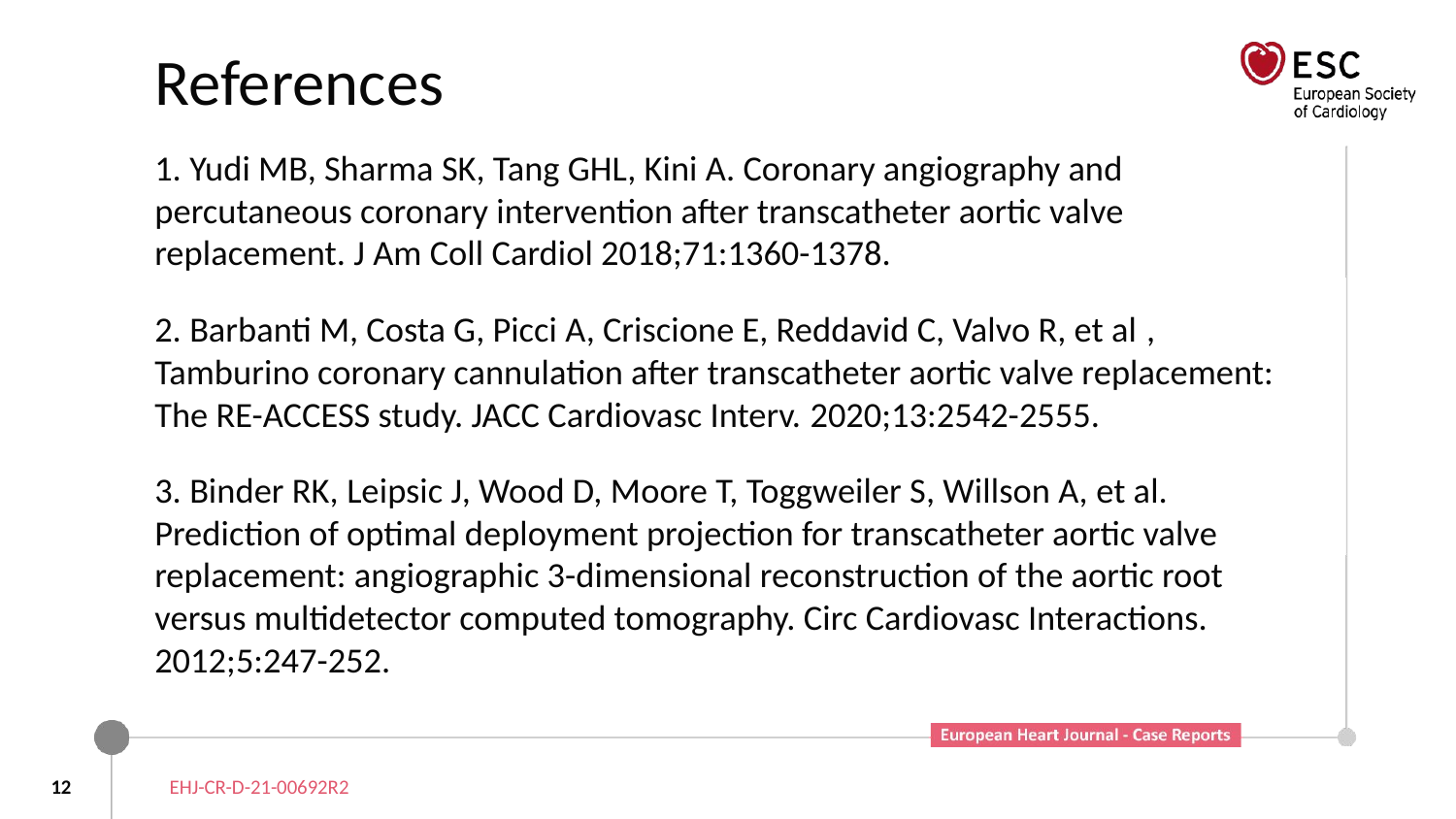

# References
1. Yudi MB, Sharma SK, Tang GHL, Kini A. Coronary angiography and percutaneous coronary intervention after transcatheter aortic valve replacement. J Am Coll Cardiol 2018;71:1360-1378.
2. Barbanti M, Costa G, Picci A, Criscione E, Reddavid C, Valvo R, et al , Tamburino coronary cannulation after transcatheter aortic valve replacement: The RE-ACCESS study. JACC Cardiovasc Interv. 2020;13:2542-2555.
3. Binder RK, Leipsic J, Wood D, Moore T, Toggweiler S, Willson A, et al. Prediction of optimal deployment projection for transcatheter aortic valve replacement: angiographic 3-dimensional reconstruction of the aortic root versus multidetector computed tomography. Circ Cardiovasc Interactions. 2012;5:247-252.
12
EHJ-CR-D-21-00692R2
